# Improved yield of canine islet isolation from deceased donors

**DOI:** 10.1186/s12917-017-1177-2

**Published:** 2017-08-22

**Authors:** Stephen Harrington, S. Janette Williams, Vern Otte, Sally Barchman, Cheryl Jones, Karthik Ramachandran, Lisa Stehno-Bittel

**Affiliations:** 1 University of Kansas, School of Engineering, Lawrence, KS 66048 USA; 2 University of Kansas Medical Center, MS 2002, Kansas City, KS 66160 USA; 3 Likarda, LLC, 2002 W 39th Avenue, Kansas City, KS 66103 USA; 4 State Line Animal Hospital, 2009 W 104th Street, Leawood, KS 66206 USA

**Keywords:** Canine diabetes, Islet isolation, Islet transplantation

## Abstract

**Background:**

Canine diabetes is a strikingly prevalent and growing disease, and yet the standard treatment of a twice-daily insulin injection is both cumbersome to pet owners and only moderately effective. Islet transplantation has been performed with repeated success in canine research models, but has unfortunately not been made available to companion animals. Standard protocols for islet isolation, developed primarily for human islet transplantation, include beating-heart organ donation, vascular perfusion of preservation solutions, specialized equipment. Unfortunately, these processes are prohibitively complex and expensive for veterinary use. The aim of the study was to develop a simplified approach for isolating canine islets that is compatible with the financial and logistical restrictions inherent to veterinary medicine for the purpose of translating islet transplantation to a clinical treatment for canine diabetes.

**Results:**

Here, we describe simplified strategies for isolating quality islets from deceased canine donors without vascular preservation and with up to 90 min of cold ischemia time. An average of more than 1500 islet equivalents per kg of donor bodyweight was obtained with a purity of 70% (*N* = 6 animals). Islets were 95% viable and responsive to glucose stimulation for a week. We found that processing only the body and tail of the pancreas increased isolation efficiency without sacrificing islet total yield. Islet yield per gram of tissue increased from 773 to 1868 islet equivalents when the head of the pancreas was discarded (*N* = 3/group).

**Conclusions:**

In summary, this study resulted in the development of an efficient and readily accessible method for obtaining viable and functional canine islets from deceased donors. These strategies provide an ethical means for obtaining donor islets.

## Background

Historically dogs have played an important role in the field of diabetes research. From the discovery of insulin from pancreatic extracts of dogs [1], to the use of dogs in research for islet transplants, canines have been viewed as an appropriate animal model, because of the similarities between human 1 type 1 diabetes and spontaneous canine diabetes. Vrabelova et al. recently published an excellent review to this very point, comparing the similarities in the disease and treatment of human and canine diabetes [2].

The isolation of canine islets has been a process developed almost entirely with laboratory animals under optimal, controlled conditions, i.e. beating-heart donors, minimal periods of ischemia, specialized equipment, and little regard to cost. However, these ideal conditions for islet isolation are simply not feasible in all situations. Furthermore, the use of purpose-bred dogs for on-site, beating-heart pancreas procurement is both costly and regarded as highly unethical. An untapped alternative is donor pancreata from already euthanized donors at veterinary clinics through organ donation programs, and then transported to a separate facility for processing. Consequently, there is a clear need for a simple and cost effective strategy to obtain quality islets from euthanized donors where ischemic injury is inevitable. Recently, some promising results were reported using euthanized canine donors, but islet yields were relatively low and inconsistent [3]. Further, the method utilized a costly Ricordi chamber and complex temperature controlled perfusion system. Thus, continued improvements in this space will be useful.

In the present study, we sought to develop a simple, scalable, and cost-effective method that could be used to consistently procure large quantities of transplant-quality canine islets from euthanized donors using standard laboratory equipment and materials. To accomplish this, we first developed a custom cold preservation solution and an advanced density gradient medium using readily available components. We also administered heparin prior to euthanasia and incorporated a simple ductal purging step to eliminate residual blood in lieu of more complex vascular preservation and flushing. Finally, because the canine pancreas is known to have a higher concentration of islet tissue in the tail region [4, 5], we evaluated the implications of omitting the head of the pancreas from the isolation process on overall islet yield, purity, viability and function.

## Methods

### Organ preservation solution

A modified Histidine-Tryptophan-Ketoglutarate (HTK) organ preservation solution was prepared in house with 5% (*w*/*v*) polyethylene glycol (PEG), average M.W. 8000, added as an oncotic agent to minimize cellular edema [6–9]. The modified HTK-PEG solution was sterilized by filtration through a 0.22 μm filter unit, and the pH and density was measured. The density of the HTK-PEG preservation solution was measured using a Densito 30PX densitometer (Mettler Toledo) approximately 1.022 g/mL at 20 °C. The solution pH was 7.2-7.4 at 4 °C with a theoretical osmolality of approximately 315 mOsm/kg.

### Digestion buffer

Pancreas digestion was carried out in a custom buffer of Hank’s Balanced Salt Solution (HBSS) containing 10 mM HEPES and 3.2 mM calcium chloride, adjusted to pH 7.6-7.8 at room temperature [10–12]. This digestion buffer was also used to prepare collagenase solutions.

### Collagenase solution

A highly purified collagenase/thermolysin blend, Liberase T-Flex, (Roche Custom Biotech, Indianapolis, IN) was dissolved in the above-described HBSS digestion buffer. Enzyme solutions were prepared immediately prior to use at 3 Wunsch Units (WU) of collagenase and 0.018 mg thermolysin per mL of buffer. Sufficient volume of enzyme solution was prepared for each individual pancreas to achieve approximately 10 WU per gram of digested tissue.

### High Osmolality Iodixanol/HTK density gradient medium

A custom density gradient medium was prepared by first adding 5 M sodium chloride to a concentrated solution of iodixanol (OptiPrep, CosmoBio USA) to a final calculated osmolality of 700 mOsm/kg and a density of 1.315 g/mL at 20 °C, as measured with a Densito 30PX densitometer (Mettler Toledo). This high osmolality iodixanol stock was then mixed with the modified HTK-PEG preservation solution to create solutions of various desired densities [13]. The calculated osmolality of the final gradient media was approximately 450 mOsm/kg [14–17].

### Pancreas donors

Pancreata were obtained from canine donors at local veterinary clinics with consent of the owners for organ donation. Animals were already scheduled for euthanasia for other reasons and had no known endocrine disorders. Euthanasia was performed by the licensed veterinarian overseeing the care of each animal, and the clinical veterinarian confirmed death with loss of heart function. Collection of the donor pancreata after death, and from animals euthanized for reasons other than tissue procurement, was determined to be exempt from IACUC review by the University of Kansas Medical Center. Canine donor and procurement information for organs is provided in Table 1.

**Table 1:** Pancreas donor information

Animal ID	Breed	Gender	Age(years)	BW(kg)	WIT(minutes)	CIT(minutes)
Whole Pancreas						
W1	Lab/Pitbull	M	N/A	21	17	90
W2	Hound	M	2	25	7	86
W3	Pitbull Mix	M	2	20	13	72
Average	-	-	-	22.0	12.3	82.7
SE	-	-	-	1.7	2.9	5.5
Tail and Body						
TB1	Shepherd Mix	M	1	21	23	75
TB2	Hound	M	2	31	10	72
TB3	Wheaton Terrier	M	2	20	23	87
Average	-	-	-	24.0	18.7	78.0
SE	-	-	-	3.5	4.3	4.6
No Heparin/Ductal Flush						
NH1	Pitbull Mix	M	0.5	10	28	77
NH2	Pitbull/Shar Pei Mix	F	2	23	25	72
NH3	Shepherd Mix	F	5	20	20	90
NH4	Chow Mix	M	4	10	20	150
Average	-	-	-	15.9	23.2	97.2
SE	-	-	-	3.3	2.0	18.0
Histological Study						
IHC1	Boxer	F	8	27	20	85
Combined Average (All Donors)	-	-	-	20.7	18.7	86.9
SE	-	-	-	1.9	1.9	6.7

*Abbreviations*: *W* whole pancreas, *TB* tail and body, *NH* no heparin/ductal flush, *IHC* immunohistochemistry, *N/A* not available, *BW* bodyweight, *WIT* warm ischemia time, *CIT* cold ischemia time, *SE* standard error

Donor pancreata were categorized into four experimental groups: whole pancreas (W), tail and body (TB), no heparin/ductal flush (NH), and histological study (IHC). The IHC group was not used for pancreas digestion or islet isolation.

### Pancreas procurement and preservation

Prior to euthanasia, donors were given heparin sulfate intravenously at 300 units/kg with the exception of the NH study group. After cardiac death, the abdominal fur was shaved, and the surface sanitized with Povidone-Iodine sponges (Medline Industries, Inc.) and rinsed with 70% ethanol. Then, the abdominal cavity was accessed via gross midline incision and a small lateral incision near the stomach. The major splenic and pancreaticoduodenal vessels leading into the pancreas were ligated and the duodenum was double clamped with large hemostats on either side of the pancreas. The major vessels were severed above the ligatures to minimize blood contamination as the pancreas was removed with the attached duodenal section. The isolated pancreas was then immersed in cold HTK-PEG preservation solution and transported to the laboratory for processing.

### Tissue processing for histological studies

In order to confirm in a non-research bred animal, the well-characterized differences in islet distribution throughout the pancreas, described in research-bred beagles, a single organ donor was utilized for histology (donor ICH1, Table 1). The pancreas was manually dissected into three parts (head, middle and tail). The tissues were fixed using the general methods we have previously described [18]. Samples were placed in 10% normal buffered formalin in phosphate buffered saline (PBS), pH 7.2, for 3 days at +4 °C. Tissues were embedded in paraffin using an automated vacuum tissue processor Leica ASP300S (Leica Microsystems Inc. Bannockburn, IL) and stored at +4 °C. The samples were sectioned in 7 μm thicknesses using a microtome RM2255 (Leica Microsystems) and mounted directly on Superfrost/Plus microscope slides (Fisher, Pittsburgh, PA). After cutting, the slides were dried overnight at +40 °C in an oven and stored at +4 °C until processed.

Paraffin embedded sections were deparaffinized and subsequently dehydrated in xylene followed by ethanol and PBS serial rehydration. Antigen retrieval was completed in a steamer using 0.01 M citrate buffer, pH 6.2, with 0.002 M EDTA, for 30 min. After cooling for 20 min, slides were washed in PBS 2 times and permeabilized in1% Triton X-100 in PBS for 30 min, and subsequently rinsed in PBS. After washing, sections were encircled with a PAP pen. Sections were incubated in 10% normal donkey serum, 1% BSA, 0.03% Triton X-100, all diluted in PBS, for 30 min to block nonspecific binding sites and rinsed by dipping in PBS once. Blocked sections were used for immunofluorescence and immunohistochemistry staining.

### Immunofluorescence

The immunofluorescence procedures have been described by our group previously [18]. Blocked sections were incubated with a primary antibody mix at +4 °C, overnight, in a wet chamber. The following primary antibodies were used to stain the pancreas: anti-insulin (1:400, Abcam, Cambridge, MA, #ab7842), anti-glucagon (1:400, Abcam, #ab10988), anti-somatostatin (1:400, Abcam, #ab53165). Sections were rinsed in PBS 3 times, 10 min each, and incubated for 2 h at room temperature in a mix of fluorophore-conjugated secondary antibodies in wet chamber protected from light. Appropriate secondary antibodies were used that were conjugated with DyLight 488 (1:400, Jackson ImmunoResearch Laboratories Inc., West Grove, PA), Alexa 555 (1:400, Molecular Probes, Eugene, OR), or Alexa 647 (1:400, Molecular Probes). The same solution was used to dilute primary and secondary antibodies: 1% NDS, 1% BSA, 0.03% Triton X-100. After incubation with secondary antibody, slides were washed in PBS 3 times, 10 min each, and mounted with anti-fading agent Gel/Mount (Biomeda, Foster City, CA). Insulin, somatostatin, and glucagon were labelled by green, blue, and red fluorescent probes, respectively.

Stained slides were viewed using a Nikon C1Si microscope. Images were captured on a Nikon C1Si or C1 Plus confocal microscope, and were analyzed using Nikon software EZ-C1 3.90 Free viewer.

### Immunohistochemistry

Immunohistochemistry procedures have been described previously [18]. Anti-insulin (1:200, Santa Cruz Biotechnology, Inc**.,** Santa Cruz, CA, #sc9168), anti-glucagon (1:200, Santa Cruz Biotechnology, #sc13091), or anti-somatostatin (1:200, Abcam, #ab15365) primary antibodies were used for pancreas immunostaining. Staining was developed using Histostain-*Plus* Broad Spectrum (AEC) Kit (Invitrogen, Frederick, MD). Slides were counterstained with hematoxylin to identify cell nuclei.

After staining, slides were rinsed in deionized water and placed on coverslips in Clear Mount mounting medium (Electron Microscopy Sciences, Hatfield, PA). The specificity of immunoreactivity was confirmed by omitting the primary antibody from some sections. The staining was observed using a light microscope Nikon Eclipse 80i (Nikon Instruments, Melville, NY). Images were analyzed using Adobe Photoshop CZ4 extended software.

### Pancreas digestion

For organs used to isolate islets, the pancreas was placed on a cooled sterile surgical tray for processing. The attached duodenal section was resected from the pancreas and discarded, and the mass of the pancreas was recorded. The accessory pancreatic duct was located and cannulated using a sterile 18 G, 20 G, or 22 G catheter, depending on the size of the ductal lumen [3]. Six pancreata (groups W and TB, Table 1) underwent a preliminary saline ductal flush and rinse. These pancreata were completely distended with saline via the accessory duct and immersed in cold sterile saline solution to purge the organ of excess blood. In order to examine the effectiveness of the ductal purging technique, four pancreata did not undergo a preliminary ductal saline flush and rinse (group NH, Table 1). Heparin was not administered in these donors. All pancreata were next perfused with freshly prepared collagenase solution (described above) using a 30 mL syringe and flexible Luer extension set. Phenol red dye was included in the enzyme buffer to visually confirm the complete perfusion of enzyme throughout the gland. Heparinized and flushed pancreata were further divided into two groups. In the first group (group W, Table 1), the whole pancreas was filled with collagenase and processed. In the second group (group TB, Table 1), the head of the pancreas was not perfused by ensuring the catheter was only in the main tail/body branch, and subsequently ligating the head of the pancreas with a large hemostat. The unfilled pancreas head was then removed, weighed and discarded. The digested tissue mass was recorded for each isolation.

Following enzyme perfusion, pancreata were cut into 2–3 cm pieces and divided into 250 mL polycarbonate flasks containing ten silicon nitride beads for mechanical disruption. Each 250 mL flask contained a maximum of 25 g of tissue. Flasks were then filled with additional cold digestion buffer to a total volume of ~250 mL. Finally, the flasks were placed in a 38 °C shaking water bath at 120 rpm with occasional manual shaking. During the digestion process, small samples of the tissue were stained with dithizone to evaluate the extent of islet liberation from exocrine tissue. When at least 50% of islets were free of exocrine, or no improvement in islet liberation was observed in subsequent samples, digestion was stopped by transferring the tissue into a 500 mL centrifuge bottle containing 250 mL cold RPMI 1640 media with 10% bovine calf serum (BCS). Bottles were placed on ice and the tissue was allowed to settle by gravity for 15 min. The supernatant was discarded and the tissue was transferred to 225 mL centrifuge tubes, rinsed with additional RPMI with 10% BCS, and centrifuged at 400 rcf (relative centrifugal force) for 5 min at 4 °C [3].

### Islet purification

Tissue pellets were re-suspended in cold HTK-PEG solution and triturated several times with a 25 mL serological pipette and then a 30 mL syringe. The tissue was passed through a 500 μm steel mesh to remove large, undigested debris [19]. The tissue digest was collected in clean 225 mL centrifuge tubes and pelleted at 400 rcf for 5 min at 4 °C and the supernatant discarded. Pellets were dried by inverting the tubes on sterile gauze for 60 s and then weighed. The tissue was re-suspended in HTK-PEG solution at an approximate concentration of 0.1 g per mL and stored at 4 °C for a minimum of 45 min [20, 21].

After the cold incubation period, an initial test gradient purification was performed to verify the appropriate density for islet purification. Fifteen mL of tissue/HTK-PEG suspension was combined with 6.5 mL of the high osmolality iodixanol stock to bring the density of the medium to 1.110 g/mL and incubated on ice for 5 min. The tissue digest was deposited beneath 25 mL of RPMI with 10% BCS in a 50 mL centrifuge tube to generate a two-layer discontinuous gradient. The gradient was centrifuged at 400 rcf for 5 min at 4 °C. The entire supernatant (containing purified islets) was poured in a clean tube and centrifuged again while the pellet (containing exocrine tissue) was discarded. The second pellet, which contained the purified islets, was evaluated by dithizone staining for exocrine contamination before processing the remaining tissue. If necessary, the density of the gradient medium was modified in order to achieve a minimum target islet purity of 60%.

Islet purity was evaluated by visual inspection at 24 h after isolation under a light microscope using dithizone stain to distinguish islets from non-islet tissues [22] Dithizone stain (Sigma Aldrich) was prepared by dissolving dithizone in dimethyl sulfoxide (DMSO) at 10 mg/mL, and then diluting with PBS to a concentration of 0.2 mg/mL, and was added to islet suspensions at 10% *v*/v. To obtain purity, five representative samples from each donor were stained and examined via bright field microscopy. Islet purity was estimated to the nearest 5% for each sample and the values were averaged to obtain the final purity. Isolations with islet purity below 60% were considered unacceptable, and were not used for further studies.

### Islet culture

Isolated canine islets were cultured in 150 mm petri dishes in 25 mL of CMRL 1066 media (with a glucose concentration of 5.6 mM) supplemented with 10% fetal bovine serum, 2 mM glutamine (GIBCO**®** GlutaMAX, ThermoFisher Scientific), and an antibiotic-antimycotic (GIBCO**®** Anti-Anti 100X, ThermoFisher Scientific) at 37 **°**C and 5% CO_2_ at a maximum density of 5000 islet equivalents per dish [23, 24]. Media was exchanged the morning after isolation followed by every other day at a minimum of 50% by volume.

### Islet yield assessment

Islet yields of each isolation were calculated according to the standard method of conversion to islets equivalents, or “IEQ”, which accounts for the size variation of native islets [25]. Due to the size disparity of canine donors, islet yield was also calculated per gram of digested tissue and per kilogram of donor bodyweight.

### Viability assessment

Islet viability was evaluated using a live/dead fluorescence assay. Islets in PBS were stained with calcein AM and PI at 4 μM and 1 μg/mL, respectively and imaged by fluorescence microscopy [24, 26]. Dithizone stain (described above) was added at 10% *v*/v to distinguish islets from exocrine and ductal tissue, which were not included in the viability calculations. Fluorescence images were analyzed using Adobe Photoshop CC. The percentage of dead cells was obtained by dividing the number of red pixels (propidium iodide) per islet by the total islet pixels. Data are reported as percent viability. Viability was assessed between 24 and 72 h after isolation. Twenty-five individual islets per isolation were analyzed to determine the percent viability.

### Glucose stimulated insulin secretion via static incubation

Glucose stimulated insulin secretion (GSIS) of the isolated canine islets was evaluated by static incubation of islets in buffers containing varying levels of glucose. All glucose solutions were made in an EBSS (Earl’s Balanced Salt Solution) buffer with 0.1% BSA and sodium bicarbonate added, pH 7.4 at 37 °C [23]. All incubation steps were completed at 37 **°**C at 5% CO_2_. First, islets were equilibrated to the basal medium of 2.8 mM glucose for 1 h. Using Transwell inserts (8.0 um pore size) in a 24-well plate, approximately 50 IEQ were transferred to a low glucose condition of 2.8 mM glucose, followed by 22.4 mM (high) glucose, and finally 30 mM KCl, a standard insulin secretagogue [27] for 90 min each. The Transwell procedure allows the same islets to be exposed to the different glucose concentrations, thus nullifying the need for normalization to IEQ/well. Supernatant media was collected after low, high, and KCl incubations and stored at −80 **°**C until insulin quantification was performed. GSIS was assessed in triplicate for islets from a total of four individual donors (2 tail/body and 2 whole pancreas) after 3 and 7 days of culture.

Insulin concentration was determined with alphaLISA insulin assays (Perkin Elmer, Waltham, MA) and a EnSpire® plate reader (Perkin Elmer) equipped with the corresponding alpha technology. Assays were carried out according to the manufacturer’s instructions with insulin standards fit to a 12-point curve fit to a 5-parameter logistic curve. Insulin concentration data were then used to calculate stimulation indices, which is the ratio of insulin secretion in high glucose to low glucose.

### Statistical analysis

Pancreas digestion times were evaluated using a one-tailed student’s t-test to determine if the heparin and saline flush treatment resulted in significantly reduced digestion times compared to the untreated group. Pairwise comparisons for islet yield per gram of tissue were done using a one-tailed student’s t-test to determine whether IEQ per gram of tissue was significantly higher in the tail/body vs. the whole pancreas groups. Pairwise comparisons for donor bodyweight, warm ischemia time, cold ischemia time, IEQ per donor bodyweight, islet viability, islet purity, and stimulation indices were evaluated for significance differences via two-tailed student’s t-test. Significance was defined in all cases as *p* < 0.05.

## Results

### Effect of pre-mortem heparin and Ductal saline flush

**Fig. 1:**
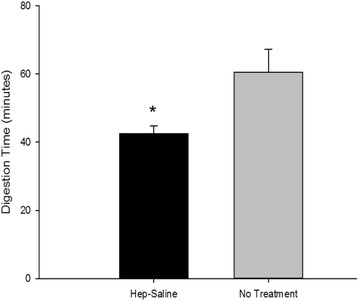
Effect of heparin and ductal flush. The effects of pre-mortem heparin and post-mortem ductal saline flushing steps on enzymatic digestion were evaluated. Digestion times were reduced significantly when these steps were incorporated (* denotes significance, *p* = 0.01; *N* = 6 for the Hep-Saline group and *n* = 4 for the no treatment group)

The method reported herein incorporated two additional steps to enhance enzymatic digestion of the pancreas compared to previously reported procedures [3, 28, 29]. First, heparin sulfate was administered pre-mortem to prevent blood coagulation within the pancreatic tissue after euthanasia. Second, the pancreas was distended with cold saline solution and then rinsed by immersion in cold saline followed by enzymatic perfusion. We detected no significant difference in donor bodyweight, warm ischemia time, or cold ischemia time between the heparinized and non-heparinized donors (Table 1). Digestion time was significantly reduced when heparin and saline treatments were included compared to the untreated group (Fig. 1). Pancreatic tissues digested quickly and uniformly after heparin and ductal saline flush treatments. Without treatment, the pancreatic digest contained larger and more granular particles that were unable to pass through a 500 μm screen, indicating incomplete and non-uniform digestion. Furthermore, isolation results obtained from the untreated pancreata were considered unacceptable for further evaluation because they did not meet our minimum criteria of 60% islet purity.

### Regional distribution of endocrine cells in canine pancreas

**Fig. 2:**
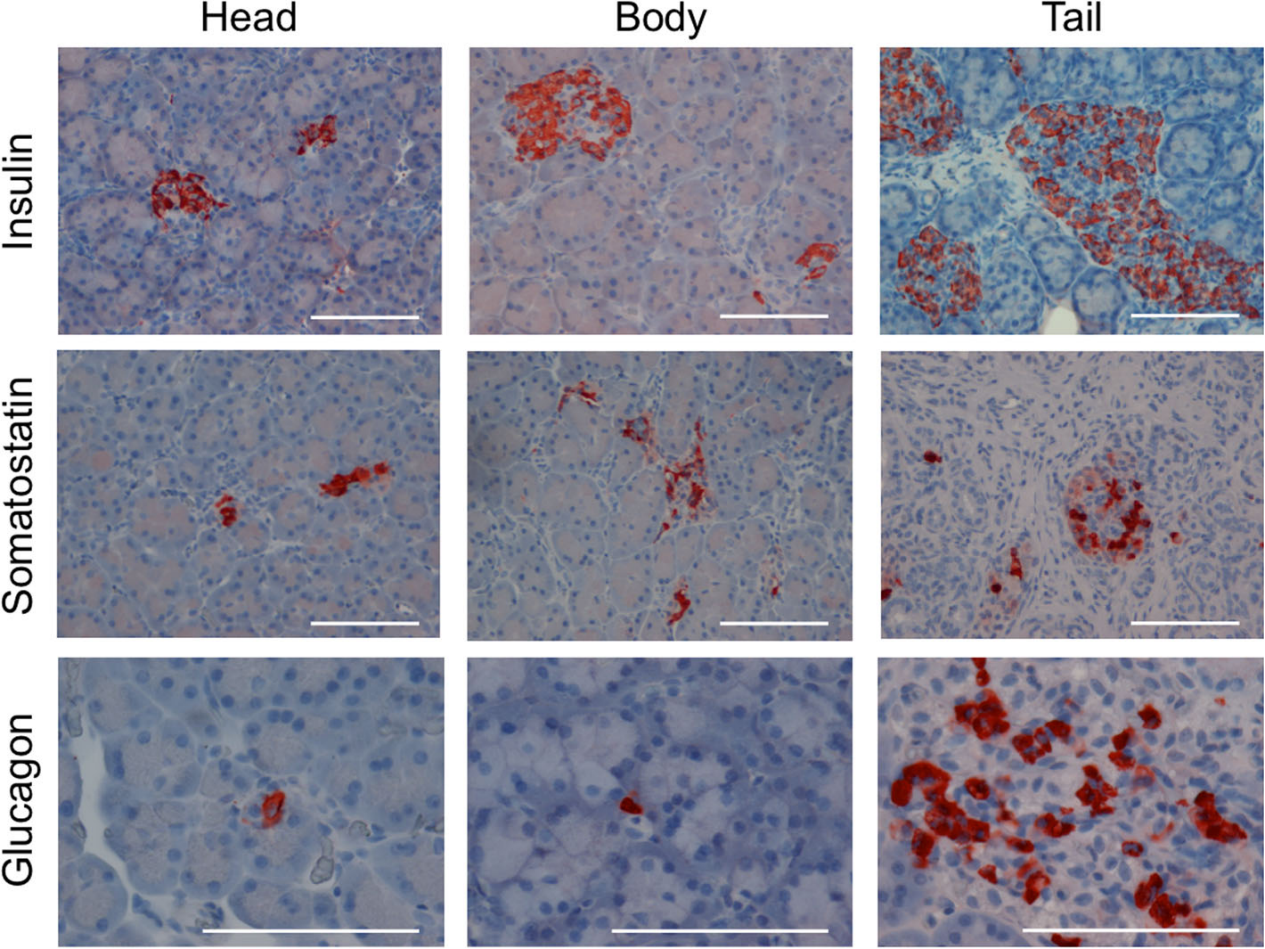
Immunohistochemistry of canine pancreas by region. Tissue sections of the three major regions of the pancreas were stained with antibodies for insulin (top row), somatostatin (center row), and glucagon (bottom row) to identify the three major islet cell types (beta, delta, and alpha cells, respectively). Insulin positive cells were primarily located in the body (center column) and tail (right column), with the highest concentration and largest sized clusters in the tail. Insulin positive cells in the head of the pancreas (left column) were far less prevalent and occurred primarily in very small clusters or single cells. Similar patterns were observed for somatostatin, while glucagon positive cells were present in extremely low numbers in the head and body and often appeared as isolated single cells. Glucagon images are displayed at higher magnification to better illustrate single cells. *Scale bars* = 200 μm

The distribution of islets within the pancreas is not uniform in the dog, as reported previously [5, 30]. Specifically, the body and tail of the canine pancreas has been reported to contain more glucagon and insulin-positive cells, indicating more islet tissue compared to the head of the pancreas [4, 31]. However, those studies focused on research-bred animals. To confirm findings in a non-research dog, we repeated the procedure on a single animal using immunohistochemistry and immunofluorescence. Figure 2 illustrates typical images from canine pancreatic sections from the head, body and tail of the pancreas. Insulin staining was less frequently noted in the head compared to other sections of the pancreas, and was most abundant in the tail region. The same was true of glucagon and somatostatin. However, glucagon-positive cells were rare in both the head and body sections, and were often found surrounded by the appearance of acinar cells rather than other islet cells as shown in Fig. 2.

**Fig. 3:**
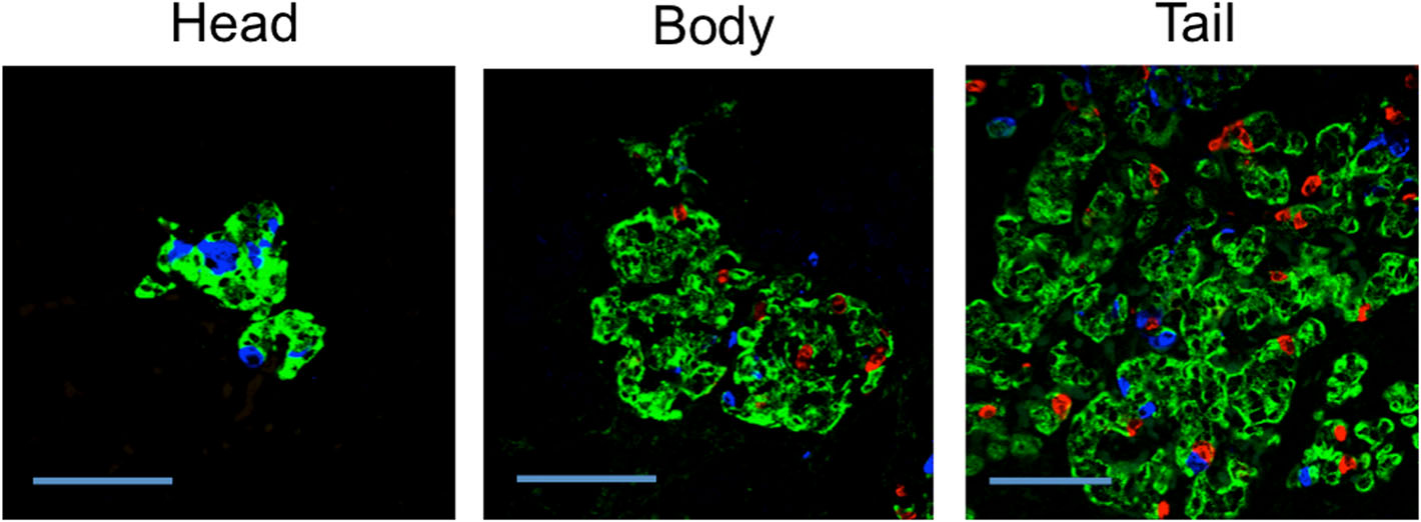
Immunofluorescence of canine pancreas by region. Pancreatic sections were triple-stained with fluorescent antibodies for insulin (green), glucagon (red), and somatostatin (blue) to identify variations in the clustering patterns of pancreatic cell types within different regions of the pancreas. Interestingly, glucagon-positive cells were rarely observed in the head of the pancreas, and were not associated with insulin- and somatostatin-positive cells. Insulin- and somatostatin-positive cells were observed within single clusters in the head region, which were typically comprised of only a few cells. In contrast, the tail and body region contained structures consisting of large numbers of cells that predominantly contained all three endocrine cell types, with the highest concentration and largest clusters of cells located in the tail region. *Scale bars* = 100 μm

To further identify whether glucagon cells were located independently of the islet structures in the canine pancreatic head, body and tail, triple-labeled immunofluorescence was employed. Typical images are shown in Fig. 3 from each region where insulin, somatostatin, and glucagon are labelled by green, blue, and red fluorescent probes, respectively. In the head, groups of insulin- or somatostatin-positive cells with glucagon positive cells were not observed. In fact, there were almost no glucagon-positive cells noted in the head of the pancreas in general, corresponding to the results noted using immunohistochemistry (Fig. 2). The body and tail both displayed larger islets with a mixture of insulin-, glucagon-, and somatostatin-positive cells. In the body and tail, all three cell types were found both in islet structures and as independent cells scattered through the exocrine tissue.

### Effect of pancreas region on yield of islets

**Fig. 4:**
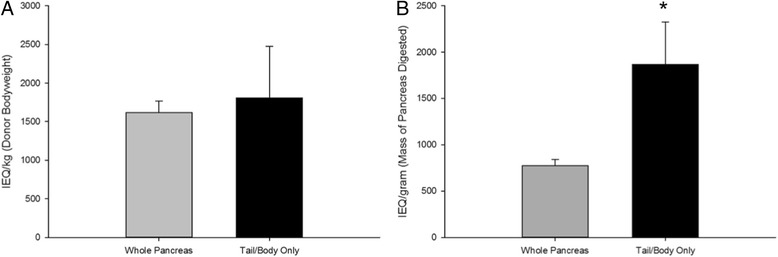
Effect of pancreas region on islet yield. **a** The effect of processing only the tail and body of the pancreas were evaluated. No significant difference in islet yield was observed when the whole pancreas was processed versus only the tail and body. IEQ values were normalized to donor bodyweight (per kg) to account for the high variability of canine donor size. **b** Islet yield per gram of tissue digested was significantly improved by omitting the head of the pancreas from processing (* denotes significance, *p* = 0.04; *N* = 3 per animals/group)

Due to the uneven distribution of islets within the pancreas, we conducted studies to determine whether exclusion of the head of the pancreas would statistically decrease the total islet yield per pancreas. Pancreata were divided into two groups for the regional islet isolation studies (W and TB), as shown in Table 1. In group W, the whole pancreas was digested, while only the tail and body of the pancreas were digested in group TB. There were no significant differences in the warm or cold ischemia times or donor bodyweights between the two groups (W vs TB, Table 1). Table 2 summarizes the isolation outcomes for the two groups, and illustrates that the total tissue mass digested in the whole pancreas group was nearly double that in the tail and body group. When the average islet yield (IEQ) was normalized to donor bodyweight, there was no significant difference in the total islet yield between the two groups (Fig. 4a). However, when the islet yield was normalized to the actual mass of pancreas digested, isolation from only the tail/body region did not decrease the yield of islets (Fig. 4b).

**Table 2:** Islet isolation outcomes by pancreas region

Animal ID	Tissue Mass Digested (g)	Total IEQ	Purity (%)
Whole Pancreas			
W1	40	29,014	76
W2	57	39,134	63
W3	40	36,283	73
Average	45.7	34,810	70.7
SE	5.7	3012	3.9
Tail and Body Only			
TB1	12	16,964	67
TB2	35	49,335	74
TB3	23	62,626	74
Average	23.3	42,975	71.7
SE	6.6	13,559	2.3
Combined Average (All Isolations)	34.5	38,892	71.2
SE	6.3	6474	2.1

*Abbreviations*: *W* whole pancreas, *TB* tail and body, *IEQ* islet equivalents, *SE* standard error

### Islet purity, morphology, and viability

**Fig. 5:**
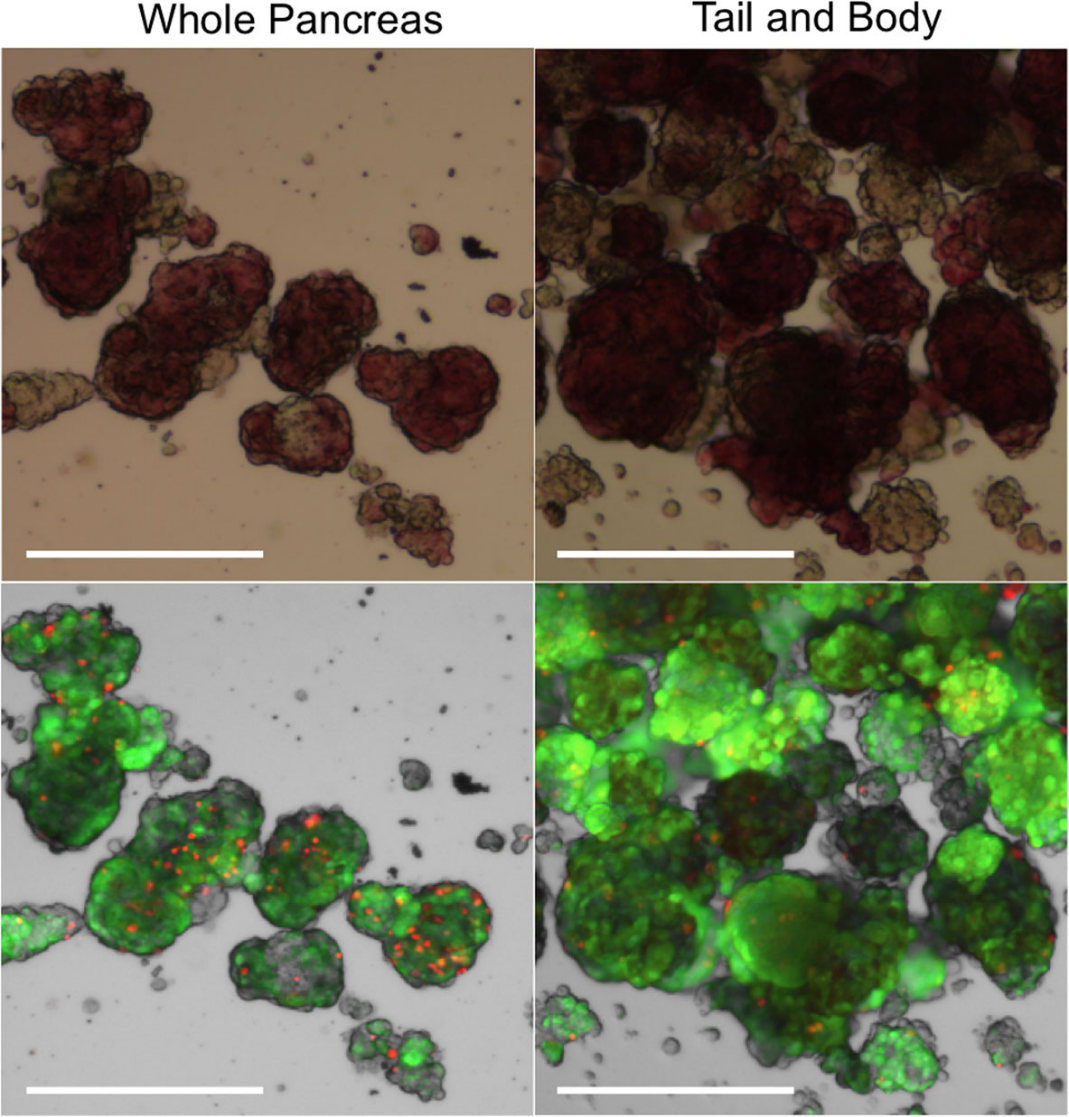
Morphology and viability of isolated islets. Morphology and viability of isolated islets were evaluated. Islets isolated from both groups (whole pancreas or tail/body only) had healthy capsules and stained deep red with dithizone, which binds to insulin within the islet (*top* row). Islets were co-stained with a live (*green* fluorescence) and dead (*red* fluorescence) stain (bottom row). Islets showed strong *green* fluorescence and sparse *red* fluorescence, indicating highly viable islets. Scale bars = 200 μm

While yield was greater per gram of tissue when only processing the body and tail of the pancreas, the final purity of each preparation was not different between the two approaches. Dithizone staining was used to distinguish between islet and non-islet tissues. Representative bright field images are shown in Fig. 5 *(top row)* **.** Islets from both groups displayed typical morphology, indicated by smooth, round edges and strong red dithizone staining. Table 2 shows that the percentage of islet cells in each preparation was approximately 70%, regardless of the starting tissue.

Islet viability was evaluated after isolation via a fluorescent live/dead assay with a representative image shown in Fig. 5 *(bottom row)* **.** Green fluorescence staining within the islet clusters indicated live cells, and red fluorescence identified dead or dying cells. The average islet viability for the whole pancreas isolations was 95.5 +/− 0.60% and for the body and tail was 95.4 +/− 0.2%. The results demonstrate very good viability of isolated islets from both groups and no significant difference between them.

### Glucose stimulated insulin secretion

**Fig. 6:**
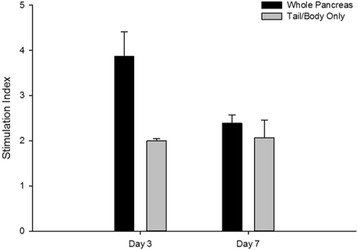
Glucose stimulated insulin secretion of isolated islets. Islet function was evaluated over time via glucose stimulated insulin secretion assay. Islets were exposed to low then high glucose, and the secreted insulin in each condition was quantified to calculate a stimulation index, or SI (high secretion/low secretion). SI’s were calculated separately for tail/body isolations and for whole pancreas isolations. Differences in SIs between the groups were not significant at either time point (*N* = 2 per group)

Islet function was evaluated via glucose stimulated insulin secretion. The same islets were first incubated in low (2.8 mM) and then high glucose (22.4 mM) and the secreted insulin in each condition was quantified and normalized to the IEQ. Secretion indices (SI), or the ratio of secreted insulin in high glucose to that in low glucose, were measured at 3, and 7 days post isolation. Figure 6 displays SI values for the whole pancreas and tail and body groups. Analysis detected no statistical differences in secretion index between the groups at either time point. Secretion indices were near or equal to 2 for all measurements, demonstrating good secretory function of the islets in both groups.

## Discussion

In this series of studies, we describe a combination of techniques for optimizing and simplifying canine islet isolation from sub-optimal donor sources. Pancreatic tissues obtained from off-site cadaveric donors will inevitably be exposed to prolonged warm and cold ischemia. Unfortunately, all of these factors are known to markedly diminish islet yield and quality [32–35].

Islets are purified via density gradient centrifugation, exploiting their lighter native density compared to pancreatic exocrine cells. Periods of warm or cold ischemia cause significant edema (swelling) of the pancreatic tissue, which alters the cellular density and thus diminishes isolation quality [34, 36]. The extended ischemia times inherent to off-site, post-euthanasia pancreas procurement exacerbate this cellular swelling, often to a point where successful isolation of islets is impossible. Fortunately, numerous strategies have already been employed in human islet isolation research to prevent and even reverse ischemic damage, generally through the use of colloid-containing organ preservation solutions and hyperosmotic density gradient media [37–42]. However, these strategies have been applied primarily to beating-heart human donor pancreata processed with specialized islet isolation equipment, with few comparisons to canine tissue under the sub-optimal conditions described above.

For these studies, we utilized a modified HTK preservation solution containing polyethylene glycol (PEG) as a colloid, because PEG is known to have many protective effects against cellular swelling and ischemic injury in general [43–45]. Further, there is emerging evidence of the benefits of PEG for islet isolation and transplantation specifically [7, 46]. Then, we added a hyperosmotic iodixanol stock to this modified HTK solution to create a custom, easily adjustable density gradient solution at approximately 450 mOsm/kg. Mildly hypertonic environments between 400 and 500 mOsm/kg have been shown to selectively increase exocrine tissue density over islets, thus enhancing the purification process [14–17, 47]. With the benefits of these readily accessible materials, we were able to achieve high quality islet isolations without the use of the Ricordi chamber and other complex, specialized equipment.

Vascular flushing has become standard practice for human pancreas preservation for islet isolation, but requires extensive training, and increases the time and cost of pancreas procurement substantially [9, 40, 41]. As such, this technique is not well suited for veterinary applications. In lieu of traditional vascular flushing, we incorporated a pre-mortem heparin injection followed by a ductal purging step directly prior to enzyme perfusion. Further, the treatment reduced the average digestion times by 18 min per pancreas, while improving isolation outcomes. These results are well in line with previous reports that shorter digestion times strongly correlate to both higher numbers of islets recovered per pancreas as well as IEQs per gram [48].

In contrast to humans, islet cell distribution and islet morphology are highly disproportionate throughout the canine pancreas. Wieczorek et al. showed that the majority of islet cells were located in the left lobe (i.e. tail and body) and were large and compact [5]. Conversely, the right lobe (i.e. head) contained mostly single cells and small islets, along with pancreatic polypeptide cells. The limited histological evaluations described here are supported by previous studies [4, 30, 31]. Thus, we evaluated the effect of completely omitting the head of the pancreas in islet isolation with the long-term goal of maintaining islet yield while reducing isolation costs. Islet yield per gram essentially doubled when only the tail and body were used while viabilty, purity, and function of the islets were not affected. Furthermore, the exclusion of the pancreas head from the isolation process did not significantly impact the total islet yield per pancreas.

To our knowledge, this is only the second report of successful islet isolation from deceased canine donors [3], and the first to do so without the use of the Ricordi system and with up to 90 min of cold ischemia time. Islets isolated by our method were 95% viable, had good purity and were responsive to glucose stimulation. We obtained an average of 1868 IEQ/g of digested pancreas (tail/body), which compares favorably to the median of 608 IEQ/g reported by Vrabelova et al. [3]. Woolcott et al. reported yields of approximately 3600 IEQ/g, but pancreata in these studies were obtained from heart-beating donors with no reported cold ischemia [29]. Similarly, Lakey et al. reported a range of 3800 – 4490 IEQ/g using a comparable enzyme blend, but again used heart-beating donors and did not report any periods of cold ischemia [49]. Unfortunately, these ideal experimental conditions are likely unattainable for veterinary applications, and would raise considerable ethical concerns. In contrast, our method, which utilized deceased donors with extended periods of warm and cold ischemia, acheived yields of greater than 1500 IEQ/kg of donor bodyweight. Generally, between 5000 and 10,000 IEQ per kg are needed to reverse diabetes in canines [50–54]. Thus, our method could obtain, from a single 35 kg canine donor, enough islets to reverse diabetes in a 10 kg recipient.

A major shortcoming of the study is the small number of donor pancreas/group. When utilizing donors from veterinary clinics rather than research-bred dogs, qualified donors can be scarce, and there are ethical implications. For example, during the study we quickly determined that the heparinization/ductal flush was a superior process. Rather than continuing with an inferior procedure, we felt it was ethically important to apply the new technique to all subsequent donor pancreas even though a small number of the non-flush procedures had been conducted. In spite of these limitations, we were able to reach statistically significant differences between groups when examing the important outcome measures of digestion time and yield. However, the authors acknowledge that the results may not be translatable to all situations. For example, there may be certain breeds in which the head of the pancreas contains a higher percentage of mature islets. Finally, while the in vitro characterizations of islets obtained by our method were promising, the authors also acknowlege the importance of in vivo transplant studies to confirm the capacity of the islets to revserse diabetes, and intend to conduct this work in future studies.

## Conclusions

In conclusion, we have demonstrated a simple method for isolating quality canine islets from deceased donors. Furthermore, our findings, in corroboration with previous histological studies [4, 5, 30, 31], suggest that the head of the canine pancreas contains negligible recoverable islet tissue, and may be safely omitted from the isolation process. However, the authors acknowledge the limitation of small sample sizes in this study along with the high level of variability associated with islet isolation, and that efforts to improve upon and further validate these processes should continue.
